# Transforming Growth Factor-β1 Induces Concurrent Periostin and SLUG Expression in Normal Human Dermal Fibroblasts: Potential Implications for Extracellular Matrix Remodeling

**DOI:** 10.3390/biomedicines14092042

**Published:** 2026-09-11

**Authors:** Patrycja Sputa-Grzegrzolka, Anna Socha-Banasiak, Natalia Glatzel-Plucinska, Mateusz Olbromski, Katarzyna Ratajczak-Wielgomas, Tomasz Gornicki, Elzbieta Czkwianianc, Piotr Dziegiel, Bartosz Kempisty

**Affiliations:** 1 Division of Anatomy, Department of Human Morphology and Embryology, Faculty of Medicine, Wroclaw Medical University, 50-368 Wroclaw, Poland; 2 Department of Gastroenterology, Allergology and Pediatrics, Polish Mother’s Memorial Hospital-Research Institute, 93-338 Lodz, Poland; 3 Division of Histology and Embryology, Department of Human Morphology and Embryology, Faculty of Medicine, Wroclaw Medical University, 50-368 Wroclaw, Poland; 4 Physiology Graduate Faculty, North Carolina State University, Raleigh, NC 27607, USA; 5 Institute of Veterinary Medicine, Department of Veterinary Surgery, Nicolaus Copernicus University, 87-100 Torun, Poland; 6 Department of Stem Cells and Regenerative Medicine, Institute of Natural Fibres and Medicinal Plants, 60-630 Poznan, Poland

**Keywords:** TGF-β1, periostin, *POSTN*, SLUG, *SNAI2*, fibroblasts, extracellular matrix, fibrosis, tissue remodeling, profibrotic signaling

## Abstract

**Background**: Transforming growth factor beta 1 (TGF-β1) is a master regulator of fibrogenesis and extracellular matrix (ECM) remodeling. Among molecules involved in profibrotic signaling, periostin (*POSTN*) and the Snail family transcriptional repressor 2 (SLUG, *SNAI2*) have emerged as potential regulators of tissue remodeling and fibroblast activation. However, the temporal relationship between TGF-β1-induced expression of SLUG and periostin in human dermal fibroblasts remains poorly understood. **Objectives**: This study aimed to evaluate the effect of TGF-β1 stimulation on *SNAI2*/SLUG and *POSTN*/periostin expression dynamics in normal human dermal fibroblasts (NHDFs) and characterize their association with profibrotic responses. **Methods**: NHDFs were stimulated with recombinant human TGF-β1 (20 ng/mL) for 24, 48, and 72 h. *SNAI2* and *POSTN* mRNA expression was quantified by RT-qPCR, intracellular SLUG protein levels were assessed by Western blotting, and periostin accumulation in the culture medium was measured by enzyme-linked immunosorbent assay (ELISA). The RT-qPCR and ELISA data were analyzed using two-way ANOVA with Bonferroni correction; because of the small number of replicates (*n* = 2), the Western blot data are presented descriptively. **Results**: TGF-β1 significantly increased *POSTN* mRNA expression after 24 and 48 h (both *p* < 0.001); at 72 h, *POSTN* mRNA levels remained numerically elevated relative to the control, although this difference did not reach statistical significance. Periostin accumulation in the culture medium increased progressively and was significantly higher than in the control group after 48 and 72 h (*p* < 0.001). In contrast, *SNAI2*/SLUG exhibited transient expression kinetics, with increased *SNAI2* mRNA at 48 h (*p* < 0.05) and the highest SLUG protein levels also observed after 48 h of stimulation, followed by a decline at 72 h. **Conclusions**: TGF-β1 induced time-dependent changes in *SNAI2*/SLUG and *POSTN*/periostin expression in human dermal fibroblasts. As fibroblast activation and ECM remodeling were not directly assessed, the present conclusions are restricted to the expression changes in the two investigated molecules, whose concurrent yet temporally distinct regulation may indicate potential relevance in the fibroblast response to profibrotic signaling. Further studies are required to determine whether they participate in common regulatory mechanisms or independently contribute to fibrotic processes.

## 1. Introduction

Chronic inflammation is one of the key factors accompanying the processes that lead to tissue remodeling and fibrosis in a wide range of organ-specific diseases. Its course is characterized by multifaceted interactions among immune cells, stromal cells, and components of the extracellular matrix (ECM), resulting in the activation of multiple signaling pathways that promote the persistence of inflammatory responses and the initiation of dysregulated reparative processes. In the context of tissue remodeling, particular importance has been attributed to fibroblast activation and their transition toward a myofibroblast phenotype, which is associated with an increased capacity for ECM production and the progression of fibrotic processes [1,2,3,4].

Among the mediators frequently investigated in the context of tissue remodeling, transforming growth factor beta (TGF-β) is considered one of the principal regulators of the fibrogenic response. TGF-β can activate both canonical and non-canonical signaling pathways that influence the expression of genes involved in tissue remodeling and stromal cell activation, including transcription factors such as SLUG (*SNAI2*) [5,6,7,8]. SLUG is widely described in the literature as a regulator of epithelial–mesenchymal transition (EMT), where it participates in the repression of epithelial marker expression and the acquisition of mesenchymal phenotypic characteristics [9,10,11,12]. At the same time, its expression and activity have also been observed in other cellular contexts associated with inflammatory signaling and tissue remodeling, indicative of a more generalized functional role beyond the classical EMT model [10,13].

Under conditions of chronic inflammation, increased TGF-β expression and nuclear localization of SLUG have been observed in fibrotic tissues, which may indicate their involvement in tissue remodeling processes [14].

Growing evidence also highlights the involvement of periostin, an extracellular matrix protein encoded by the *POSTN* gene, in inflammatory and fibrotic processes [9,15,16]. Periostin is involved in the regulation of cell adhesion, proliferation, and differentiation and may interact with integrins to modulate intracellular signaling pathways and ECM organization [17,18]. Increased periostin expression has been reported in chronic inflammatory diseases, including inflammatory bowel disease, where it has been associated with enhanced tissue remodeling and the presence of fibrosis [9,16,19,20,21].

In the context of fibrogenesis, the co-occurrence and potential interplay among TGF-β, SLUG (*SNAI2*), and periostin (*POSTN*) have been increasingly investigated [9,22,23,24]. TGF-β is widely recognized as an important mediator of fibroblast activation and ECM remodeling, in part through activation of the SMAD2/3 signaling pathway, which may induce changes in the expression of genes associated with a profibrotic phenotype [25]. In experimental models, TGF-β stimulation has been associated with increased SLUG expression, which may contribute to the regulation of genes involved in cytoskeletal reorganization and functional changes in stromal cells [26].

Concurrently, increased periostin expression has been observed in response to TGF-β stimulation in numerous experimental models, pointing to its role in extracellular matrix remodeling, fibroblast activation, and the deposition of ECM components, including collagen [27,28]. Both in vitro and in vivo studies indicate that the activation of the TGF-β/SMAD3 pathway may be associated with enhanced *POSTN* transcription, while elevated periostin expression is often observed in parallel with increased expression of markers such as α-smooth muscle actin (α-SMA) and type I collagen [29,30].

In addition, periostin has been described not only as an effector molecule but also as a protein capable of influencing the tissue microenvironment, including through modulation of fibroblast activity and SMAD-dependent signaling. Such interactions have been proposed to contribute to the maintenance of fibrotic responses under conditions of chronic inflammation. In several experimental models, silencing of periostin expression was associated with reduced SMAD2/3 pathway activation and decreased expression of fibrosis-related genes, which underscores the likely contribution of periostin to the regulation of these processes [31,32,33].

The available literature has reported the co-occurrence of increased expression of TGF-β, SLUG, and periostin in tissues affected by chronic inflammation and remodeling, including studies of skin, liver, and lung fibrosis, as well as chronic inflammatory diseases of mucosal tissues. These observations indicate that these factors likely constitute components of concurrent and potentially interconnected regulatory processes involved in fibroblast activation and ECM remodeling. However, the nature of these relationships remains incompletely understood and requires further investigation [15,29,31,34,35,36].

## 2. Materials and Methods

### 2.1. Primary Human Dermal Fibroblasts

Normal human dermal fibroblasts (NHDF-Ad-Der Fibroblasts, catalog no. CC-2511; Lonza, Basel, Switzerland) were obtained from a single donor: a 59-year-old woman of Armenoid racial ancestry (lot no. 0000693503). The cells were cultured in FBM^®^ Basal Medium, supplemented with 0.1% insulin, 0.1% recombinant human fibroblast growth factor-B (hFGF-B), 0.1% GA-1000, and 2% fetal bovine serum. Culture conditions were as follows: temperature 37 °C and 5% CO_2_ concentration. All experiments were performed using cells between passages 12 and 14. Cell cultures were routinely screened for mycoplasma contamination on a monthly basis; although the results of these tests were not systematically archived, only cultures confirmed to be free of mycoplasma contamination were used for the experiments.

All biological replicates used in this study represent independent cell cultures derived from the same donor (the same vial lot): three biological replicates were used for the RT-qPCR and ELISA experiments, and two for the Western blot analyses. To evaluate the effect of TGF-β1 on the mRNA and protein expression levels of the *POSTN*/periostin and *SNAI2*/SLUG genes, gene expression was analyzed over a 0–24–48–72 h time course.

### 2.2. In Vitro TGF-β1 Stimulation of NHDFs

To induce a profibrotic response, NHDF cells were seeded at a density of 2 × 10^5^ cells per well in 6-well plates and maintained for 24 h in complete culture medium. Subsequently, the medium was replaced with fresh medium, and the cells were treated with 20 ng/mL TGF-β1 (Merck, Darmstadt, Germany) and cultured for up to 72 h.

Every 24 h, cell lysates were collected for downstream analyses. Cell morphology was monitored by light microscopy; however, since fibroblast activation was not independently validated in this study, these observations were not used as a formal readout of activation. TGF-β1 was administered as a single dose at the start of the treatment (t = 0 h); the culture medium was not replaced, and TGF-β1 was not replenished during the 72 h incubation. In separate control experiments performed under identical treatment conditions, TGF-β1 treatment for 72 h did not significantly affect cell viability or proliferation compared with untreated controls (viability remained above 95% in all conditions).

### 2.3. RNA Extraction, cDNA Synthesis, and Real-Time PCR

Total RNA was isolated from NHDF cells using the RNeasy Mini Kit (Qiagen, Venlo, The Netherlands), according to the manufacturer’s instructions. The purity of the isolated RNA was assessed using a NanoDrop spectrophotometer (Thermo Fisher Scientific, Waltham, MA, USA). Samples with A260/A280 absorbance ratios ranging from 1.8 to 2.1 and A260/A230 absorbance ratios ranging from 1.8 to 2.2 were considered suitable for further analysis. RNA integrity was not assessed. For each sample, 500 ng of total RNA was reverse-transcribed into cDNA using the iScript™ Reverse Transcription Supermix for RT-qPCR (Bio-Rad, Hercules, CA, USA) and the C1000 Touch Thermal Cycler (Bio-Rad). Reverse transcription was performed under the following conditions: priming at 25 °C for 5 min, reverse transcription at 46 °C for 20 min, and reverse transcriptase inactivation at 95 °C for 1 min.

Quantitative real-time PCR (RT-qPCR) was performed to determine the relative expression levels of *POSTN* and *SNAI2* mRNA in NHDF cells before and after TGF-β1 stimulation. The reactions were carried out using the 7500 Real-Time PCR System (Applied Biosystems, Foster City, CA, USA) and iTaq™ Universal Probes Supermix (Bio-Rad), according to the manufacturers’ protocols. The TaqMan Gene Expression Assays used in the study were purchased from Thermo Fisher Scientific (Waltham, MA, USA) and are listed in Table 1.

All reactions were performed in three independent biological replicates, each analyzed in technical triplicate. Thermal cycling conditions consisted of an initial denaturation step at 94 °C for 2 min, followed by 45 cycles of denaturation at 94 °C for 15 s and annealing/extension at 60 °C for 1 min. The TaqMan Gene Expression Assays used in the study were pre-designed and bioinformatically validated by the manufacturer to achieve near-100% efficiency under standard cycling conditions; reaction efficiency was therefore not independently assessed. For each assay, a no-template control was included, in which water was used instead of the cDNA template. Cq values lower than 30 cycles were considered positive detection. During data analysis, variations of up to 0.5 cycles between technical triplicates were considered acceptable. For each biological replicate, the untreated control sample collected at the beginning of the experiment (0 h) was used as the calibrator, and its relative expression level was set to 1; expression of all other samples was calculated relative to this calibrator using the ΔΔCt method, with 18S rRNA (18SRNA) used as an endogenous reference gene for normalization. Of note, absolute Cq values of the analyzed genes, including 18S, declined with increasing passage number, and expression profiles varied markedly between successive biological replicates; a formal stability assessment of the reference gene (e.g., by geNorm or NormFinder analysis) could therefore not be performed, and normalization was based on within-replicate comparison against the calibrator.

### 2.4. Protein Extraction, SDS-PAGE, and Western Blotting

Membranes were blocked for 1 h at room temperature in 4% BSA in TBST and incubated overnight at 4 °C with a rabbit polyclonal anti-SLUG/SNAI2 antibody (Thermo Fisher Scientific, #PA5-73015; dilution 1:200), raised against amino acids 82–110 of human SNAI2. The working dilution was established during optimization of the assay under our laboratory conditions. Although the manufacturer reports a tested Western blot dilution of 1:2000, the relatively small amount of biological material available for these experiments—the number of cells collected at individual experimental time points was limited, resulting in a low amount of protein lysate—necessitated the use of a higher antibody concentration to obtain adequate detection of SLUG. After washing, membranes were incubated for 1 h at room temperature with HRP-conjugated secondary antibodies (e.g., donkey anti-rabbit IgG-HRP; Jackson ImmunoResearch Laboratories, Inc., West Grove, PA, USA), diluted 1:3000. Signal detection was performed using the Immun-Star HRP Chemiluminescent Substrate Kit (Bio-Rad Laboratories, Inc., Hercules, CA, USA). All Western blot images used for densitometric analysis were acquired using identical imaging settings with an exposure time of 30 s; differences in band intensity were therefore not caused by differences in exposure time. To ensure equal protein loading, membranes were reprobed with anti-β-actin monoclonal antibody (#4970; dilution 1:1000; Cell Signaling Technology, Danvers, MA, USA). Densitometric analysis was performed using the ChemiDoc™ MP Imaging System with Image Lab 5.0 software (Bio-Rad Laboratories, Inc., Hercules, CA, USA) and was restricted to the SLUG-immunoreactive band migrating at approximately 30 kDa, between the 25 and 37 kDa bands of the Precision Plus Protein Dual Color Standards (Bio-Rad, #1610374), consistent with the expected molecular mass of SLUG/SNAI2. The additional immunoreactive bands visible on the uncropped membrane were not included in the densitometric analysis; we cannot exclude the possibility that the relatively high antibody concentration contributed to their appearance. SLUG intensity was normalized to the corresponding β-actin signal from the same lane. Some lane-to-lane variation in β-actin intensity is visible in the original blots; this was accounted for by normalizing each SLUG signal to its corresponding β-actin signal rather than comparing raw SLUG intensities. Full uncropped membrane images from both biological replicates are provided in Supplementary Figure S1, together with the molecular-weight marker positions and the band used for densitometric analysis. Individual normalized densitometric values for both biological replicates (*n* = 2) (SLUG and β-actin intensity readings, SLUG/β-actin ratios, mean values, and values relative to the control) are provided in Supplementary Table S1.

### 2.5. ELISA Method

POSTN concentrations were determined in three independent biological replicates for each experimental group using a RayBio^®^ Human Periostin ELISA Kit (Catalog #: ELH-POSTN; RayBiotech, Peachtree Corners, GA, USA) according to the manufacturer’s instructions. Periostin protein concentrations were determined according to the manufacturer’s instructions. Diluted cell culture supernatants (100 μL) or standard curve samples (100 μL) were added to the wells. To assess *POSTN* concentrations, samples were incubated for 2.5 h at room temperature (RT) with gentle shaking.

Then, 100 μL of a biotinylated anti-human Periostin antibody was added and incubated for 1 h at room temperature. Next, streptavidin-HRP conjugate solution (100 μL) was added and incubated for 1 h at room temperature. Then, TMB substrate solution (100 μL) was applied and incubated for 30 min to induce the color reaction, which was stopped by adding the Stop Solution. The absorbance was measured using an Infinite 200 plate reader (TECAN, Männedorf, Switzerland) at 450 nm. Protein concentrations were determined using a log–log standard curve, and the data were averaged for statistical analysis.

Prior to the assay, cell culture supernatants were diluted 1:10 in sample diluent, and each sample was measured in duplicate technical replicates. The validated detection range of the ELISA kit (RayBio^®^ Human Periostin ELISA Kit, ELH-POSTN, Peachtree Corners, GA, USA) was 80 pg/mL–60 ng/mL, and all measured values fell within this range. The intra-assay coefficient of variation was <5%, and the inter-assay CV was <8%.

Because the culture medium was not replaced during the 72 h experiment and TGF-β1 was added as a single dose at t = 0 h (Section 2.2), the measured periostin concentrations reflect the cumulative accumulation of periostin in the culture supernatant over the entire incubation period rather than a time-specific secretion rate. Direct normalization of the measured concentrations to viable cell number or total cellular protein was not performed; instead, equivalence of cell mass across experimental groups was ensured by seeding all wells at an identical initial density (2 × 10^5^ cells per well) and by the control experiments demonstrating preserved viability (Section 2.2).

### 2.6. Statistical Analysis

Statistical analysis was carried out using Prism 5.0 software. The experimental unit was the individual cell culture well. Each condition (control or TGF-β1) and each time point (0, 24, 48, and 72 h) was represented by separate, independent wells that were cultured in parallel and harvested at the respective time point; time was therefore treated as a between-subjects factor rather than a repeated-measures factor. The RT-qPCR and ELISA experiments were performed in three independent biological replicates, and the Western blot analyses in two independent biological replicates; all replicates represented independent cell cultures derived from the same donor (the same vial lot; Section 2.1). For each biological replicate, technical replicates (triplicates for RT-qPCR, duplicates for ELISA) were averaged, and only these per-replicate means were entered into the statistical analysis; technical replicates were never treated as independent observations. The RT-qPCR and ELISA data were analyzed by two-way analysis of variance (two-way ANOVA) with treatment and time as fixed factors, followed by Bonferroni-corrected pairwise comparisons between the TGF-β1-stimulated and control groups at each time point. Results were considered statistically significant for *p* < 0.05. Given the small number of biological replicates per group (*n* = 3), normality and homogeneity of variances could not be reliably assessed by formal tests; two-way ANOVA was therefore chosen based on the experimental design, the relevant literature, and graphical inspection of the data. Because of the small number of replicates (*n* = 2), the Western blot data are presented descriptively. The treatment effect, time effect, and treatment × time interaction are reported in the Results (Section 3.1, Section 3.2 and Section 3.3), together with Bonferroni-adjusted *p*-values for pairwise comparisons.

## 3. Results

### 3.1. POSTN Gene Expression

**Figure 1:**
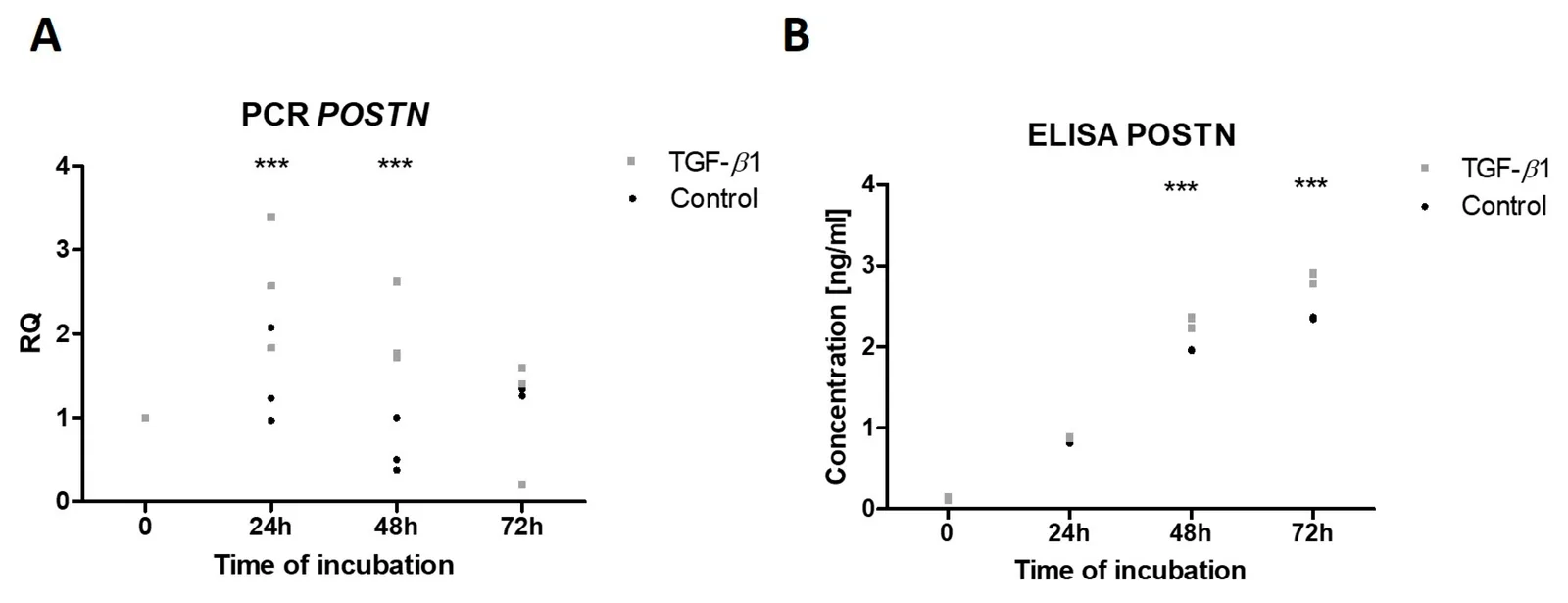
Effect of TGF-β1 on POSTN expression at the mRNA and protein levels. (**A**) Quantitative PCR (qPCR) analysis of *POSTN* mRNA expression demonstrated a significant increase following 24 h of TGF-β1 stimulation (*** *p* < 0.001), which remained elevated after 48 h of incubation (*** *p* < 0.001) compared with the control group. After 72 h, expression levels remained numerically elevated relative to the control, although this difference did not reach statistical significance. (**B**) Measurement of periostin protein concentration by ELISA revealed a gradual increase over the course of TGF-β1 incubation. Significantly higher periostin concentrations compared with the control group were observed after 48 h and 72 h (*** *p* < 0.001 for both). Individual biological replicate values (*n* = 3) for each experimental group are shown as dots. Asterisks indicate statistically significant differences between the TGF-β1-stimulated and control groups at the respective time point (two-way ANOVA with Bonferroni correction for multiple comparisons; *** *p* < 0.001). RQ—Relative Quantification.

TGF-β1 stimulation induced an increase in *POSTN* mRNA levels, reaching significantly elevated expression after 24 and 48 h of incubation (*p* < 0.001). A non-significant trend toward higher expression was also maintained at 72 h of incubation, although it did not reach statistical significance (two-way ANOVA with Bonferroni correction for multiple comparisons, Figure 1A). Two-way ANOVA confirmed a significant effect of treatment (F(1, 16) = 79.29, *p* < 0.0001), time (F(3, 16) = 22.68, *p* < 0.0001), and their interaction (F(3, 16) = 20.25, *p* = 0.0001). Bonferroni-corrected pairwise comparisons showed significant differences between the control and TGF-β-treated groups at 24 h (*p* < 0.001) and 48 h (*p* < 0.001), whereas no significant differences were observed at 0 h (*p* > 0.05) or 72 h (*p* > 0.05).

### 3.2. SNAI2 Gene Expression

**Figure 2:**
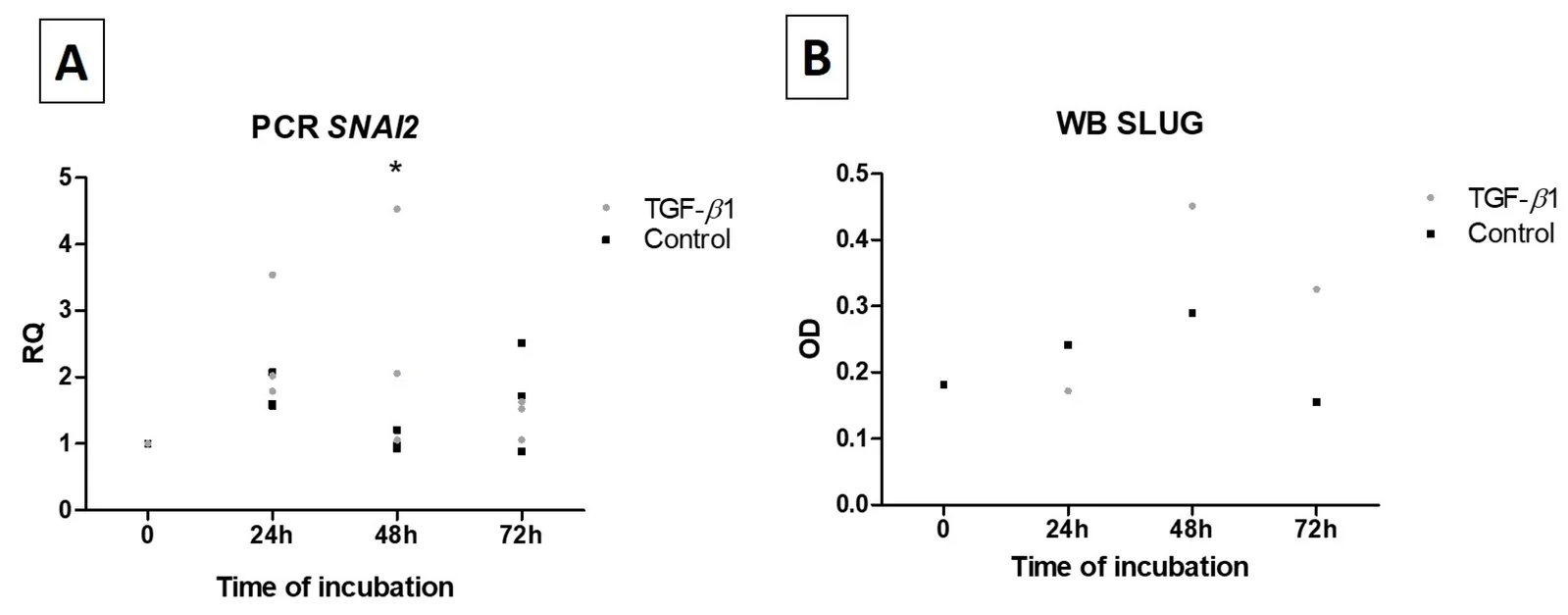
Effect of TGF-β1 on SNAI2/SLUG expression assessed by qPCR and Western blot. (**A**) qPCR analysis of *SNAI2* mRNA expression following 24 h, 48 h, and 72 h of TGF-β1 stimulation demonstrated increased expression relative to the control group, with the highest levels observed after 48 h of incubation; statistical significance was reached at 48 h (* *p* < 0.05). Individual biological replicate values (*n* = 3) for each experimental group are shown as dots; asterisks denote statistically significant differences between the TGF-β1-stimulated and control groups (* *p* < 0.05; two-way ANOVA with Bonferroni correction for multiple comparisons). (**B**) Western blot analysis of SLUG protein expression, accompanied by densitometric quantification, revealed the highest signal intensity after 48 h of TGF-β1 stimulation in both biological replicates, with no apparent increase at 24 h and a decline at 72 h, although SLUG levels remained elevated relative to the control group. RQ—Relative Quantification, OD—Optical Density.

For *SNAI2* mRNA, a trend toward increased expression was observed in NHDF cells 24 h after TGF-β1 stimulation compared to control. Similarly, at 48 h, higher SLUG mRNA expression was observed in TGF-β1-stimulated NHDF cells and reached statistical significance (*p* < 0.05). Subsequently, at 72 h, expression returned to levels comparable to those of the control group, albeit remaining minimally elevated (two-way ANOVA with Bonferroni correction for multiple comparisons, Figure 2A). Two-way ANOVA confirmed a significant effect of treatment (F(1, 16) = 8.638, *p* = 0.0097) and of time (F(3, 16) = 3.442, *p* = 0.043), but not of their interaction (F(3, 16) = 2.731, *p* = 0.078). Bonferroni-corrected pairwise comparisons showed a significant difference between the control and TGF-β-treated groups at 48 h (*p* < 0.05), whereas no significant differences were observed at 0 h (*p* > 0.05), 24 h (*p* > 0.05), or 72 h (*p* > 0.05).

### 3.3. Periostin Accumulation in the Culture Medium

ELISA measurements demonstrated a gradual increase in periostin concentration in the culture medium. In the conditioned medium of TGF-β1-stimulated NHDF cells, significantly higher periostin levels were observed compared with the control group after 48 and 72 h (*p* < 0.001; two-way ANOVA with Bonferroni correction for multiple comparisons, Figure 1B). Two-way ANOVA confirmed a significant effect of treatment (F(1, 16) = 176.7, *p* < 0.0001), time (F(3, 16) = 46.85, *p* < 0.0001), and their interaction (F(3, 16) = 55.16, *p* < 0.0001). Bonferroni-corrected pairwise comparisons showed no significant differences between the control and TGF-β-treated groups at 0 h (*p* > 0.05) or 24 h (*p* > 0.05), whereas significant differences were observed at 48 h (*p* < 0.001) and 72 h (*p* < 0.001). As the culture medium was not replaced during the experiment and TGF-β1 was added as a single dose at t = 0 h (Section 2.2), these values represent cumulative periostin accumulation in the culture supernatant over the 72 h incubation period rather than time-specific secretion rates.

### 3.4. Intracellular SLUG Protein Expression

**Figure 3:**
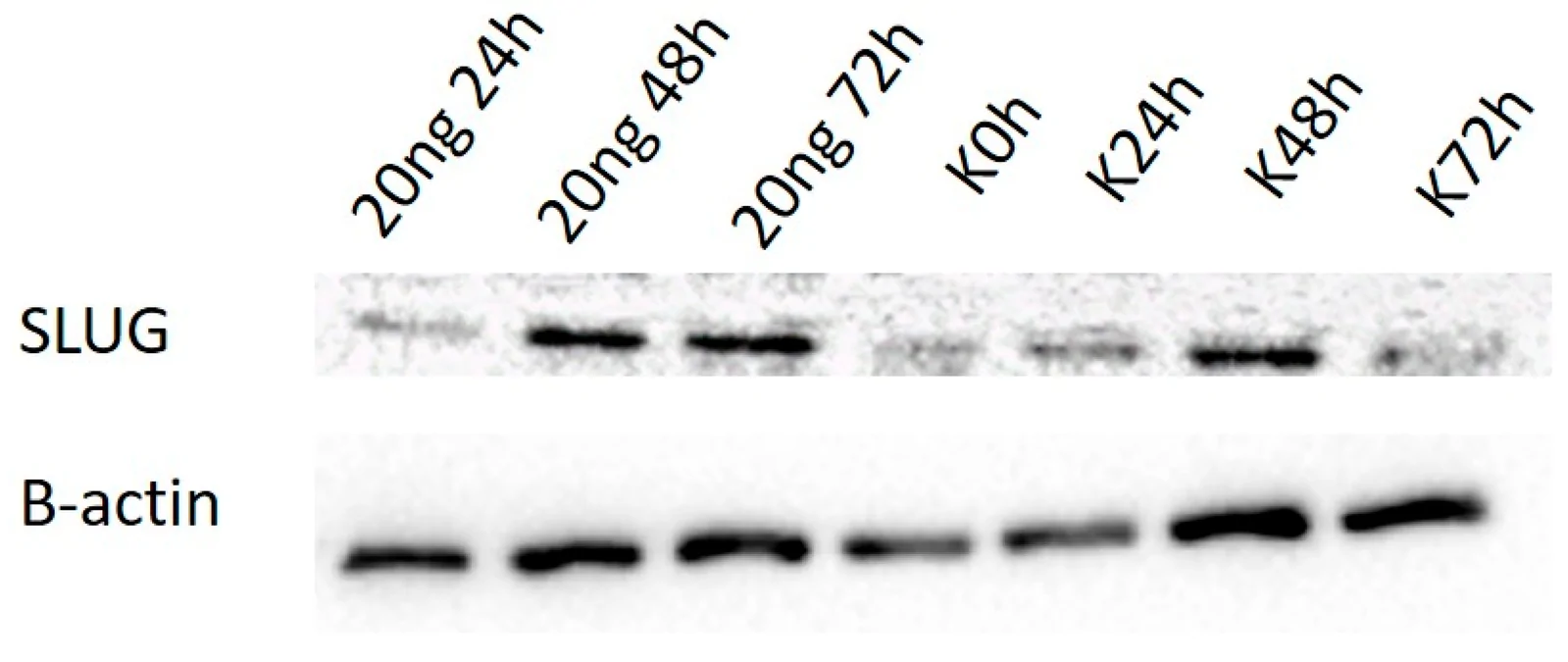
Representative Western blot images showing SLUG protein levels in cells stimulated with TGF-β1 for 24 h, 48 h, and 72 h, as well as in the corresponding control groups. β-actin was used as an internal loading control. Precision Plus Protein Dual Color Standards were used as molecular-weight markers; the quantified SLUG band migrates between the 25 and 37 kDa markers, consistent with the expected molecular mass of SNAI2/SLUG (~30 kDa). Images were acquired with identical settings and an exposure time of 30 s. The blots shown are representative of two independent biological replicates; full uncropped membrane images of both replicates, with the molecular-weight marker positions and the quantified SLUG band indicated, are provided in Supplementary Figure S1. Individual normalized densitometric values for both biological replicates (*n* = 2) are provided in Supplementary Table S1.

Densitometric analysis of Western blot bands indicated an increase in SLUG protein levels in response to TGF-β1 stimulation of NHDF cells. In both biological replicates, no increase in SLUG protein levels was observed at 24 h of incubation, with values comparable to those of the control group. The highest SLUG protein levels were observed after 48 h of incubation, reaching approximately 2.5 times the level of the untreated 0 h control (2.50 vs. 1.00), and remained elevated at 72 h (approximately 1.8-fold) despite a decline from the 48 h peak; in the control group, SLUG levels fluctuated modestly over time without a consistent increase (Figure 2B and Figure 3; Supplementary Table S1). Because the Western blot analyses were performed in only two independent biological replicates, these data were not subjected to formal statistical testing and are presented descriptively.

## 4. Discussion

In the present study, we analyzed the response of human normal dermal fibroblasts (NHDFs) to TGF-β1 stimulation in the context of connective tissue remodeling and fibrosis. We demonstrated that periostin expression in fibroblasts, both at the mRNA level (*POSTN*) and at the level of protein secreted into the culture medium in response to TGF-β1 stimulation, was significantly increased. This finding is consistent with previous observations reported by our group and other authors [9,30,34,37]. Reflecting their biological localization, the extracellular release of periostin was monitored in the culture medium by ELISA, whereas the intracellular accumulation of the transcription factor SLUG was verified in cell lysates via Western blot.

Hu Q et al., using prostate cancer cell lines, showed that TGF-β stimulation of PC3 and DU145 cells increased the expression of Twist1 and periostin, among others [27]. Similarly, Huang et al. demonstrated that periostin co-localized with activated primary mouse hepatic stellate cells (HSCs) and collagen and that TGF-β1 strongly induced its expression in these cells [37]. In turn, Nanri et al. and Naik et al., in studies on lung fibroblasts derived from patients with pulmonary fibrosis, also showed that TGF-β stimulates periostin expression in lung fibroblasts [30,34]. Additionally, Nanri et al. demonstrated that both proteins mutually amplify their activity by activating the integrin–Smad3 pathway, ultimately leading to pulmonary fibrosis [30]. However, to date, no direct association between periostin expression and the transcription factor SLUG has been established.

In the study by Sputa-Grzegrzolka et al., based on tissue material obtained from intestinal biopsies of pediatric patients with inflammatory bowel disease, a positive correlation between periostin expression and both TGF-β and SLUG expression was observed at the mRNA and protein levels [9]. Moreover, periostin and SLUG expression were mainly localized to cells within the lamina propria and submucosa, suggesting their association with activated stromal compartments during intestinal inflammation [9]. Our current findings extend these observations by demonstrating that TGF-β1 stimulation is associated with increased periostin and SLUG expression in human dermal fibroblasts in vitro. Together, these results support the potential involvement of TGF-β-dependent regulation of periostin and SLUG in fibroblast-associated profibrotic responses across different tissue contexts, although the underlying functional relationship between these molecules requires further investigation.

Our findings are consistent with the widely described role of TGF-β as a key mediator of stromal cell activation and extracellular matrix (ECM) remodeling processes [8]. It has previously been shown that activation of the TGF-β/SMAD pathway leads to increased expression of genes involved in ECM remodeling, including periostin, as well as key EMT-associated transcription factors such as SLUG (*SNAI2*), which are considered important components of the profibrotic response [37,38,39].

Periostin (*POSTN*) is widely described as a protein involved in the regulation of cell–ECM interactions, including fibroblast adhesion, migration, and matrix organization [17,18], as well as the potential consolidation of tissue architecture [14,40]. In the literature, periostin has also been linked to accelerated tissue remodeling across multiple organs, suggesting its involvement in responses to chronic inflammatory stimuli [15]. It has been proposed that periostin may influence collagen fiber organization and ECM biomechanical properties, including stiffness, which is considered one of the mechanisms sustaining the fibrotic response. Increased matrix stiffness acts synergistically with transcription factors present in the tissue niche, such as SLUG, enhancing cell activation and promoting differentiation into contractile myofibroblast-like phenotypes [17,41,42]. Dermal fibroblasts were selected for this study because they represent a well-characterized primary human fibroblast model in which TGF-β-driven profibrotic responses and periostin biology have been extensively documented, including in the context of cutaneous wound repair and systemic sclerosis [29,33]. Although the processes described above may be relevant in human pathologies such as surgically relevant intestinal strictures in IBD, reduced lung compliance in pulmonary fibrosis, or cirrhosis of the liver and kidney [6,9], findings obtained in a single dermal fibroblast model cannot be directly extrapolated to other organs and should be interpreted with caution. Furthermore, the stability of the 18S rRNA reference gene could not be formally demonstrated across the biological replicates (see Section 2.3), and the RT-qPCR results should therefore be interpreted with this limitation in mind.

An additional important aspect may be the potential positive feedback loop between TGF-β and periostin. It has been reported that TGF-β induces *POSTN* expression, while periostin itself may further enhance profibrotic signaling through integrin-mediated pathways and ECM remodeling mechanisms [43,44]. This suggests that periostin may act not only as a marker of fibroblast activation but also as an active participant in sustaining the altered tissue microenvironment [37].

The second analyzed component was the transcription factor SLUG (*SNAI2*). Although it is classically defined as a regulator of epithelial–mesenchymal transition (EMT) [11,12], increasing evidence indicates its independent role in fibroblast activation and their adaptation to chronic cytokine stimulation [10]. SLUG is involved in the direct regulation of genes responsible for cytoskeletal reorganization and migration of mesenchymal cells [13].

In our study, we observed differences in the temporal expression patterns of both markers following TGF-β1 stimulation. SLUG exhibited a transient response, with the highest levels at 48 h and a decline by 72 h, whereas periostin expression showed a more sustained increase over time, with *POSTN* mRNA already elevated at 24 h and periostin protein accumulating progressively in the culture medium. These findings may suggest that SLUG and periostin are involved in different stages or aspects of the fibroblast response to profibrotic stimulation; however, the biological significance of these temporal differences requires further investigation.

Notably, the temporal patterns of *SNAI2* mRNA and SLUG protein were broadly concordant: both were highest after 48 h of TGF-β1 stimulation. At 72 h, *SNAI2* mRNA returned toward control levels, whereas SLUG protein remained elevated relative to the control, indicating that SLUG protein abundance is not an instantaneous readout of *SNAI2* transcription. SLUG is a highly unstable protein whose steady-state levels are controlled in part at the post-translational level, including ubiquitin–proteasome-mediated degradation; the sustained protein levels at 72 h may therefore reflect an offset between residual translation and proteolytic turnover, although differences in the detection kinetics of the two assays cannot be formally excluded. Mechanistic studies assessing SLUG protein half-life and its translational or post-translational regulation will be required to clarify this temporal relationship in TGF-β1-stimulated fibroblasts. Importantly, for periostin, a clear temporal discrepancy between mRNA expression and protein accumulation was observed. This phenomenon, typical for extracellular matrix proteins, results from post-transcriptional regulation as well as the ability of periostin to be stably deposited in the extracellular environment [15,29]. It should also be noted that, as the culture medium was not replaced during the experiment, the ELISA measurements reflect cumulative periostin accumulation over the 72 h period rather than a time-specific secretion rate.

Although the study is based on a single in vitro model [3], the co-occurrence of SLUG and periostin changes in response to TGF-β1 may reflect parallel engagement of processes associated with fibroblast responses and ECM remodeling; however, since neither myofibroblast/ECM markers (e.g., α-SMA, type I collagen) nor TGF-β pathway activation (e.g., p-SMAD2/3) were assessed, fibroblast activation and ECM remodeling were not directly demonstrated in this study [27]. This relationship is further supported by in vivo pathology, where biopsy studies have revealed a strong positive correlation between SLUG expression and periostin deposition in regions of active tissue remodeling [9]. To further explore this proposed functional link, future studies should expand the experimental panel to include classical markers of myofibroblast differentiation (e.g., α-SMA, type I collagen, fibronectin, and vimentin) [29], as well as functional assays of cell contractility and migration following *SNAI2* or *POSTN* knockdown.

In summary, our data suggest that TGF-β1 promotes the expression of periostin and SLUG in human dermal fibroblasts. Although both molecules were concurrently upregulated, they displayed distinct temporal kinetics, with a transient SLUG response peaking at 48 h and a more sustained, cumulative periostin accumulation, and no direct regulatory or functional relationship was tested in this study. Accordingly, the concurrent regulation of these molecules may tentatively suggest their potential involvement in the fibroblast response to profibrotic signaling. Further studies are required to determine their functional relationship and to clarify their contribution to ECM remodeling and fibrotic processes [9].

## 5. Conclusions

1. The observed TGF-β1-induced changes in *SNAI2*/SLUG and *POSTN*/periostin expression in normal human dermal fibroblasts indicate a time-dependent response of these molecules to TGF-β1 stimulation. As fibroblast activation and ECM remodeling were not directly assessed in this study, the present conclusions are restricted to the expression changes in the two investigated molecules.
2. The concurrent, yet temporally distinct, regulation of *SNAI2*/SLUG and *POSTN*/periostin may be consistent with their involvement in related aspects of the profibrotic response; however, no direct regulatory or functional relationship was tested in this study, and whether these molecules are functionally linked remains to be determined.
3. Further mechanistic studies are required to determine whether SLUG and periostin are functionally linked within a common signaling pathway or contribute independently to fibrotic processes.
4. Given that the present findings were obtained in a single in vitro model, their potential translational value remains a working hypothesis. Whether the combined assessment of SLUG and periostin could provide a useful framework for investigating fibroblast activation or serve as a candidate biomarker in chronic inflammatory and fibrotic disorders would require validation in additional experimental models and clinical studies.

## Figures and Tables

**Table 1:** TaqMan probes used in the experiment.

Gene Product	Gene Symbol	TaqMan Probe
Periostin	*POSTN*	Hs00170815_m1
SLUG	*SNAI2*	Hs00950344_m1
18S ribosomal RNA	*18SRNA*	Hs03003631_g1

## Data Availability

The data presented in this study are available on request from the corresponding author. The data are not publicly available due to privacy issues.

## Supplementary Materials

The following supporting information can be downloaded at: https://www.mdpi.com/article/10.3390/biomedicines14092042/s1, Figure S1: Full uncropped membrane images from Western blot analysis of SLUG protein levels in NHDF cells stimulated with TGF-β1 (20 ng/mL) for 24, 48, and 72 h, together with the corresponding untreated control groups; β-actin was used as an internal loading control. The two biological replicates (*n* = 2) are shown separately. Precision Plus Protein Dual Color Standards were used as molecular-weight markers; molecular weight decreases from the top to the bottom of the membrane. The SLUG-immunoreactive band migrated at approximately 30 kDa, between the 25- and 37-kDa markers and closest to the 25-kDa marker, whereas β-actin was detected at approximately 42 kDa, between the 37- and 50-kDa markers. The SLUG bands selected for densitometric quantification are indicated. Images were acquired using identical settings with an exposure time of 30 s. Individual normalized densitometric values for both biological replicates are provided in Supplementary Table S1; Table S1: Individual densitometric values for SLUG and β-actin in Western blot analysis. Two independent biological replicates (*n* = 2) per condition are shown. Band intensities were quantified using Image Lab software (ChemiDoc™ MP Imaging System). SLUG/β-actin ratios were calculated as the quotient of SLUG and β-actin band intensities for each replicate. Mean ratios were calculated from the two biological replicates; values relative to the control were normalized to the mean ratio of the untreated 0-h control (set to 1.00).

## Abbreviations

The following abbreviations are used in this manuscript:

TGF-β1	Transforming growth factor beta 1
ECM	extracellular matrix
NHDF	Normal human dermal fibroblasts
TGF-β	Transforming growth factor beta
EMT	Epithelial-mesenchymal transition
α-SMA	α-smooth muscle actin
FBS	Fetal bovine serum
RT-qPCR	Quantitative real-time PCR
qPCR	Quantitative PCR
RQ	Relative Quantification
OD	Optical Density
HSCs	Hepatic stellate cells
