# Supplementary material for: Transforming Growth Factor-β1 Induces Concurrent Periostin and SLUG Expression in Normal Human Dermal Fibroblasts: Potential Implications for Extracellular Matrix Remodeling
Source: Biomedicines. 2026 Sep 11;14(9):2042. doi: 10.3390/biomedicines14092042 (PMC13604878; doi:10.3390/biomedicines14092042)
Supplement: Supplementary file 1 [file biomedicines-14-02042-s001.zip › biomedicines-4469172-supplementary.pdf]

Supplementary Table S1. Individual densitometric values for SLUG and β-actin in Western blot analysis. Two independent biological replicates (n = 2) per condition are shown. Band intensities were quantified using Image Lab software (ChemiDoc™ MP Imaging System). SLUG/β-actin ratios were calculated as the quotient of SLUG and β-actin band intensities for each replicate. Mean ratios were calculated from the two biological replicates; values relative to the control were normalized to the mean ratio of the untreated 0-h control (set to 1.00).

Experimental group	Time point	Biological replicate	SLUG band intensity (au)	β-actin band intensity (au)	SLUG/β-actin ratio	Mean SLUG/β-actin ratio	Relative to control (0 h = 1.00)
Control	0 h	1	197316	1015033	0.194394	0.180935	1.000
Control	0 h	2	184991	1104586	0.167475	0.180935	1.000
Control	24 h	1	299034	1190356	0.251214	0.241676	1.336
Control	24 h	2	278906	1201473	0.232137	0.241676	1.336
Control	48 h	1	506970	1684514	0.300959	0.290299	1.604
Control	48 h	2	491337	1757040	0.279639	0.290299	1.604
Control	72 h	1	223420	1452896	0.153776	0.155249	0.858
Control	72 h	2	230140	1468468	0.156721	0.155249	0.858
TGF-β1 (20 ng/mL)	24 h	1	219960	1279072	0.171968	0.171841	0.950
TGF-β1 (20 ng/mL)	24 h	2	231548	1348458	0.171713	0.171841	0.950
TGF-β1 (20 ng/mL)	48 h	1	655974	1482800	0.442389	0.451571	2.496
TGF-β1 (20 ng/mL)	48 h	2	620101	1345846	0.460752	0.451571	2.496
TGF-β1 (20 ng/mL)	72 h	1	444042	1398541	0.317504	0.325501	1.799
TGF-β1 (20 ng/mL)	72 h	2	472046	1415440	0.333498	0.325501	1.799

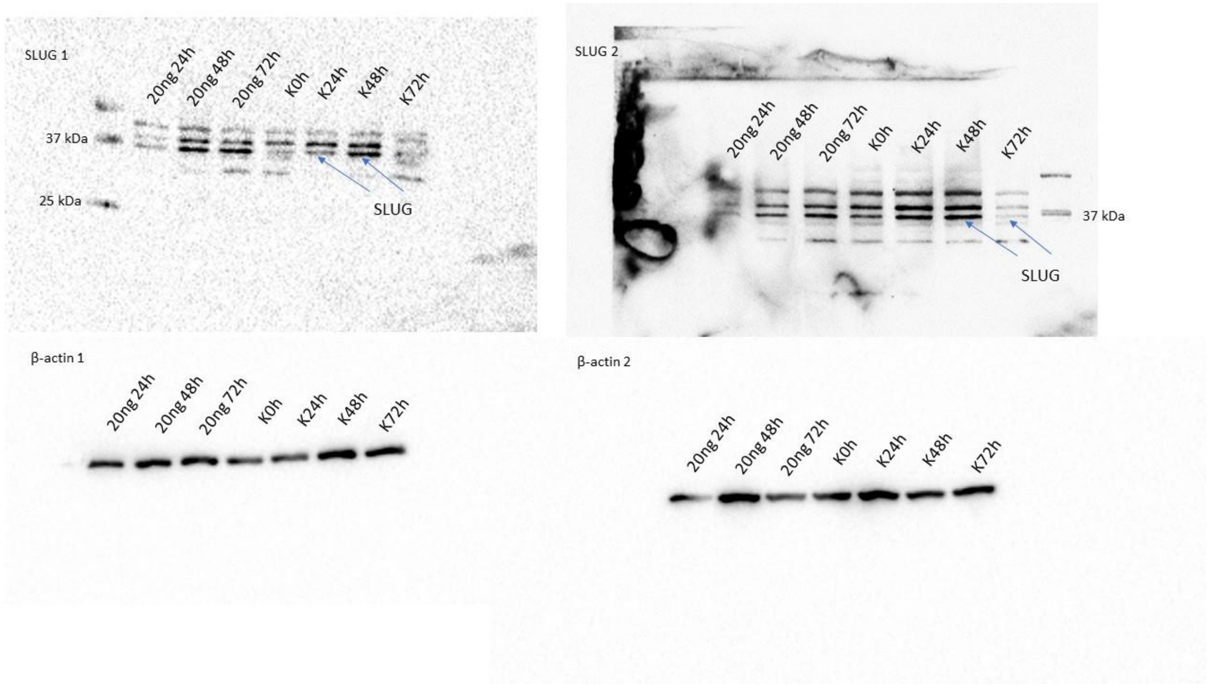

Supplementary Figure S1. Full uncropped membrane images from Western blot analysis of SLUG protein levels in NHDF cells stimulated with TGF-β1 (20 ng/mL) for 24, 48, and 72 h, together with the corresponding untreated control groups; β-actin was used as an internal loading control. The two biological replicates (n = 2) are shown separately. Precision Plus Protein Dual Color Standards were used as molecular-weight markers; molecular weight decreases from the top to the bottom of the membrane. The SLUG-immunoreactive band migrated at approximately 30 kDa, between the 25- and 37-kDa markers and closest to the 25-kDa marker, whereas β-actin was detected at approximately 42 kDa, between the 37- and 50-kDa markers. The SLUG bands selected for densitometric quantification are indicated. Images were acquired using identical settings with an exposure time of 30 s. Individual normalized densitometric values for both biological replicates are provided in Supplementary Table S1.
